# Association of the *SHOX2* and *RASSF1A* methylation levels with the pathological evolution of early-stage lung adenocarcinoma

**DOI:** 10.1186/s12885-024-12452-x

**Published:** 2024-06-05

**Authors:** Jiaping Zhao, Yu Lu, Xiaosha Ren, Tingting Bian, Jia Feng, Hui Sun, Lei Liu, Bin She, Yifei Liu, Honggang Ke

**Affiliations:** 1 Department of Thoracic Surgery, Affiliated Hospital of Nantong University, Nantong University, No.20 XISI road, ChongChuan District, NanTong, 226001 Jiangsu China; 2 Medical School of Nantong University, Nantong University, ChongChuan District, NanTong, 226001 Jiangsu China; 3 Department of Academic Development, Shanghai methyldia technology Co. Ltd, No. 412 Huiqing Road , Shanghai, 201203 China; 4 Department of Pathology, Affiliated Hospital of Nantong University, Nantong University, No.20 XISI road, ChongChuan District, NanTong, 226001 Jiangsu China; 5 Tellgen Corporation Co. Ltd, No. 115, Lane 572, Bibo Road, Pilot Free Trade Zone, Shanghai, 201203 China

**Keywords:** Lung adenocarcinoma, *SHOX2*, *RASSF1A*, Methylation, Invasiveness

## Abstract

**Background** The methylation of *SHOX2* and *RASSF1A* shows promise as a potential biomarker for the early screening of lung cancer, offering a solution to remedy the limitations of morphological diagnosis. The aim of this study is to diagnose lung adenocarcinoma by measuring the methylation levels of *SHOX2* and *RASSF1A*, and provide an accurate pathological diagnosis to predict the invasiveness of lung cancer prior to surgery.

**Material and methods** The methylation levels of *SHOX2* and *RASSF1A* were quantified using a LungMe® test kit through methylation-specific PCR (MS-PCR). The diagnostic efficacy of *SHOX2* and *RASSF1A* and the cutoff values were validated using ROC curve analysis. The hazardous factors influencing the invasiveness of lung adenocarcinoma were calculated using multiple regression.

Results: The cutoff values of *SHOX2* and *RASSF1A* were 8.3 and 12.0, respectively. The sensitivities of LungMe® in IA, MIA and AIS patients were 71.3% (122/171), 41.7% (15/36), and 16.1% (5/31) under the specificity of 94.1% (32/34) for benign lesions. Additionally, the methylation level of *SHOX2*, *RASSF1A* and LungMe® correlated with the high invasiveness of clinicopathological features, such as age, gender, tumor size, TNM stage, pathological type, pleural invasion and STAS. The tumor size, age, CTR values and LungMe® methylation levels were identified as independent hazardous factors influencing the invasiveness of lung adenocarcinoma.

Conclusion: *SHOX2* and *RASSF1A* combined methylation can be used as an early detection indicator of lung adenocarcinoma. *SHOX2* and *RASSF1A* combined (LungMe®) methylation is significantly correlated to age, gender, tumor size, TNM stage, pathological type, pleural invasion and STAS. The SHOX2 and RASSF1A methylation levels, tumor size and CTR values could predict the invasiveness of the tumor prior to surgery, thereby providing guidance for the surgical procedure.

## Introduction

Lung cancer is the leading cause of cancer deaths, and the high mortality rate of lung cancer can largely be attributed to late diagnosis, underscoring the critical importance of early diagnosis in mitigating cancer progression [1]. Currently, conventional diagnostic techniques employed for lung cancer encompass computed tomography imaging and morphological examination. The increasing sensitivity of CT imaging has facilitated the early detection of adenocarcinoma, resulting in smaller lesion sizes and fewer samples could be taken. While the implementation of screening has significantly enhanced the detection of early-stage lung cancer, it has also led to a rise in cases of overdiagnosis and overtreatment. Meanwhile, the morphological diversity of early lesions poses challenges in directly distinguishing between benign and malignant conditions. The identification of non-invasive early lung cancer or precancerous lesions imposes a significant psychological burden on patients and poses challenges for clinical surgeons in decision-making processes. Diagnosing small benign and malignant lesions poses a challenge, as does localizing malignant lesions during surgical procedures. Patients’ continuing observation presents a dilemma: on the one hand, the patient’s psychological pressure is heightened; on the other hand, the doctor is not completely sure whether the lesion is indolent or rapidly malignant progressing.

As we all know, the pathological evolution of early lung adenocarcinoma goes through the process of atypical adenomatous hyperplasia (AAH) to adenocarcinoma in situ (AIS) to micro invasive adenocarcinoma (MIA) to invasive adenocarcinoma (IA). In 2021, lung adenocarcinoma in situ (AIS) was excluded from the diagnostic category of lung adenocarcinoma by WHO and redefined as Precursor glandular lesion. Several studies have demonstrated the feasibility of sublobectomy in cases of AIS and minimal MIA. Sublobectomy, including wedge resection or segmentectomy, has been shown to maximize the preservation of lung function while ensuring the oncological effect, shorten the operation time, reduce postoperative complications, and yield favorable health and economic outcomes. The diverse imaging characteristics of lung adenocarcinoma nodules do not provide sufficient information to accurately predict histopathological features or patient prognosis. Comprehensive pathological judgment requires complete resection of the nodule. Secondly, the accurate pathological diagnosis of lung cancer poses a significant challenge in clinical practice. Conventional morphological diagnostic methods, such as cytological and histological examination, are susceptible to variations in specimen quality and the proficiency of pathologists.

The advancement of epigenetic research has garnered significant interest in the role of DNA methylation in the pathogenesis of cancer [2, 3]. Extensive research conducted in recent years has demonstrated that aberrant DNA methylation in these specific regions is a highly established epigenetic alteration in human cancers [4]. This characteristic not only holds the potential for distinguishing cancer cells from normal tissue but also presents opportunities for its application in early cancer detection.

Short Stature Homeobox 2 (*SHOX2*) methylation pattern has been employed for the purpose of diagnosing lung cancer. The expression of *SHOX2* is significantly elevated in the majority of cancer types. Through a comparative analysis of *SHOX2* methylation in lung cancer and normal tissues, it was observed that 96% of tumor tissues exhibited an elevated level of methylation [5]. Furthermore, the presence of high *SHOX2* expression or hypomethylation is indicative of inferior differentiation and an unfavorable prognosis [6]. Ras-association domain family member 1 A (*RASSF1A*), a tumor suppressor gene, is usually missing in several cancers [7]. *RASSF1A* has been extensively investigated as an adjunctive DNA methylation biomarker in the context of lung cancer. The promoter region of *RASSF1A* demonstrated hypermethylation in 63% of non-small cell lung cancer (NSCLC) cells, while remaining unaffected in normal epithelial cells [8]. Additionally, *RASSF1A* methylation level could predict the disease progression in non-small cell lung cancer patients receiving pemetrexed-based chemotherapy [9]. The combination diagnosis of *SHOX2* and *RASSF1A* has demonstrated utility in diagnosing a diverse range of tumors. To enhance its applicability in a diagnostic context, an in vitro diagnostic test kit, known as the LungMe® Assay (Tollgen, Shanghai, China), has been developed and validated for NMPA marking by the China National Medical Products Administration. The utilization of *SHOX2* and *RASSF1A* methylation has been shown to enhance the sensitivity of early lung cancer detection, as substantiated by multiple scholarly literature [10, 11]. The sensitivity of the combined methylation for *SHOX2* and *RASSF1A* in bronchoalveolar lavage fluid for NSCLC was found to range from 71.5 to 83.2%, while the specificity ranged from 90.0 to 97.4% [12, 13]. The combined promoter methylation assay for lung cancer using *SHOX2* and *RASSF1A* demonstrated a sensitivity of 89.8% and a specificity of 90.4% in FFPE specimens [14]. The objective of this research is to examine the involvement of *SHOX2* and *RASSF1A* methylation in the early detection of lung adenocarcinoma, specifically in distinguishing between AIS, MIA and IA. Additionally, the study aims to investigate the potential of *SHOX2* and *RASSF1A* methylation as a supplementary diagnostic tool for early lung adenocarcinoma cases with uncertain pathological diagnoses. Early-stage lung adenocarcinoma exhibits several invasive characteristics, such as pleural invasion, lymph node invasion, airway dissemination, and pathological subtyping, which influence the selection of surgical procedures. This study delves into the association between methylation and lung adenocarcinoma pathological evolution, with the hope that more information can be provided by methylation in diagnosing early lung adenocarcinoma to assist in the selection of surgical strategies. Therefore, there is a pressing necessity to establish a comprehensive system that integrates non-invasive imaging characteristics with minimally invasive diagnostic sampling to enhance the assessment of lung cancer invasiveness with greater precision and efficacy. This advancement will facilitate clinicians in making more informed and accurate decisions regarding disease management prior to initiating treatment.

## Materials and methods

### Patients and specimens

The FFPE resection specimens were collected from 272 patients who visited the Affiliated Hospital of Nantong University. Out of these specimens, a total of 238 cases of lung adenocarcinoma were diagnosed. This included 171 cases of invasive adenocarcinoma, 36 cases of minimally invasive adenocarcinoma, 31 cases of carcinoma in situ, and 34 cases of benign lesions that served as controls. The FFPE samples had not been stored for a duration exceeding 2 years. The age, gender, and other pertinent information of the patients were shown in Table 1. The study was approved by the Ethics Committee of Affiliated Hospital of Nantong University. All the patients/participants provided written informed consent to partake in this study.

**Table 1:** Baseline characteristics of patients

		Lung adenocarcinoma		
Clinicopathological index	BC (*n* = 34)	AIS (*n* = 31)	MIA (*n* = 36)	IA (*n* = 171)
Age (years)				
Median ± SEM	61.0 ± 9.1	57.0 ± 13.5	67.0 ± 12.5	64.0 ± 11.2
Range	44–83	24–75	30–78	26–81
Gender and Smoking				
Female (%)	8(23.5)	28(90.3)	22(61.1)	106(62.0)
Non-smoking	8	28	22	105
Smoking	0	0	0	1
Male (%)	26(76.5)	3(9.4)	14(38.9)	65(38.0)
Non-smoking	8	3	11	51
Smoking	5	0	3	14

Note AIS: Adenocarcinoma in situ; MIA: Microinvasive adenocarcinoma; IA: Invasive adenocarcinoma; BC: benign lesion

### DNA extraction and processing

The paraffin-embedded tissue material was lysed using the FFPE DNA extraction kit (CWY009S, CW Biotech Co., Ltd., China). The Qubit dsDNA HS Assay Kit (Life Technologies, Carlsbad, CA) was conducted to assess the DNA concentration on a Qubit® 3.0 fluorometer. Subsequently, 50 ng of DNA underwent sodium bisulfite treatment with the Tellgen DNA Purification Kit (Tellgen, Shanghai, China) to convert unmethylated cytosine to uracil.

### Detection of DNA methylation levels in FFPE specimens

The China National Medical Products Administration (NMPA) approved in vitro diagnostic (IVD) test LungMe® (20,173,403,354, Tellgen, Shanghai, China) was utilized to ascertain the DNA methylation levels in FFPE specimens. The bisulfite-converted DNA that had been purified was utilized directly for MS-PCR using the commercially available LungMe® Real-time PCR kit (Tellgen, Shanghai, China). The PCR process was conducted on an ABI 7500 Real-Time PCR instrument (Applied Biosystems, CA, UAS). The primers were as follows: SHOX2 F: 5’- TTGTTTTTGGGTTCGGGTT-3’, R: 5’- CATAACGTAAACGCCTATACTC-3’; RASSF1A F: 5’- CGGGGTTCGTTTTGTGGTTTC-3’, R: 5’- CCGATTAAATCCGTACTTCGC-3’. The corresponding channels of amplification of methylated *SHOX2*, *RASSF1A*, and ACTB were VIC, FAM, and CY5, respectively. The calculation of the methylation level for each specific gene was determined by employing the subsequent formula: ΔCt = Ct − Ct_β−ACTB_.

### ROC curve

Receiver operator characteristic (ROC) curves were employed to assess the diagnostic effects of *RASSF1A* and *SHOX2*. All the specimens were divided into cancer and non-cancer groups. Carcinoma in situ was classified as a precancerous lesion. Therefore, the non-cancerous group induced patients with AIS and benign lesions. The cancer group covered patients with MIA and IA. The Youden indexes and areas under the curve (AUC) were detected using the ROC curve.

### Statistical analysis

IBM SPSS Statistics 21.0 software (SPSS Inc., Chicago, IL) was conducted to perform the statistical analyses, and GraphPad Prism 8.0 was utilized to generate graphics. The ROC curve was employed to ascertain the ΔCt cutoff values of *SHOX2* and *RASSF1A*, with the objective of assessing the diagnostic efficacy. The sensitivity and specificity of methylation were evaluated in diagnosing lung adenocarcinoma. Additionally, the methylation examination and the examination of tumor imaging features and clinicopathological characteristics were analyzed using the chi-square test. The hazardous factors influencing the invasiveness of lung adenocarcinoma were calculated using multiple regression. P-value < 0.05 was deemed statistically significant.

## Results

### The methylation levels of SHOX2 and RASSF1A in surgical FFPE specimens

**Fig. 1:**
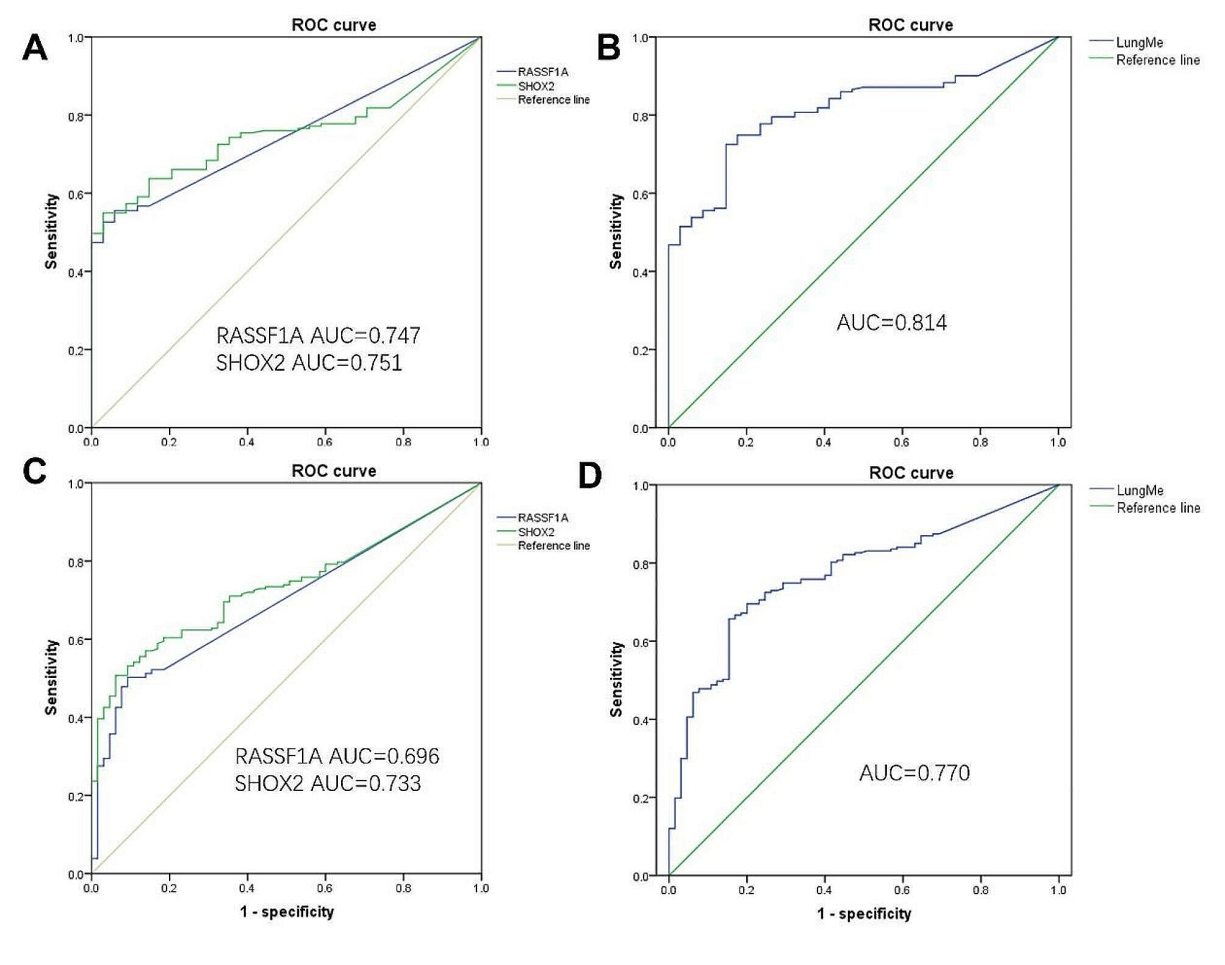
ROC curve determines the cutoff values of *SHOX2* and *RASSF1A* methylation. A: The ROC curves of *SHOX2* and *RASSF1A* methylation in distinguishing IA and BC. B: The ROC curve of LungMe® methylation in distinguishing IA and BC. C: The ROC curve of *SHOX2* and *RASSF1A* methylation in distinguishing cancer group (IA and MIA) and non-cancer groups (AIS and BC). D: The ROC curve of LungMe® methylation in distinguishing cancer group (IA and MIA) and non-cancer groups (AIS and BC)

To explore the diagnostic efficiency, the methylation levels of *SHOX2* and *RASSF1A* in surgical FFPE specimens were measured using MS-PCR. To calculate the cutoff values of *SHOX2* and *RASSF1A*, ROC curves were performed using two methods of grouping. On one hand, the patients with invasive adenocarcinoma are in the cancer group and benign lesions are in the non-cancer group. At this time, the AUCs of *SHOX2* and *RASSF1A* methylation were 0.747 and 0.75 (Fig. 1A), while the AUC of LungMe® was 0.814 (Fig. 1B). On the other hand, all the specimens were divided into cancer and non-cancer groups. Due to adenocarcinoma in situ being classified as a precancerous lesion, AIS together with benign lesions control (BC) was classified into non-cancerous group. For another, MIA and IA were classified into cancer group. Through ROC curve, the AUCs of *SHOX2* and *RASSF1A* methylation were 0.696 and 0.733 (Fig. 1C), while the AUC of LungMe® was 0.770 (Fig. 1D). The calculated cutoff values of *RASSF1A* methylation, determined by employing two distinct methods, were both found to be 13.94. However, considering our prior research, which indicated that a cutoff value exceeding 12.0 resulted in inadequate PCR stability, we decided to modify the cutoff value to 12.0. For the cutoff of *SHOX2* methylation, the calculated cutoff values of *SHOX2* using the two methods were 8.27 and 8.28, respectively. Ultimately, we opted for the rounded value of 8.3.

**Fig. 2:**
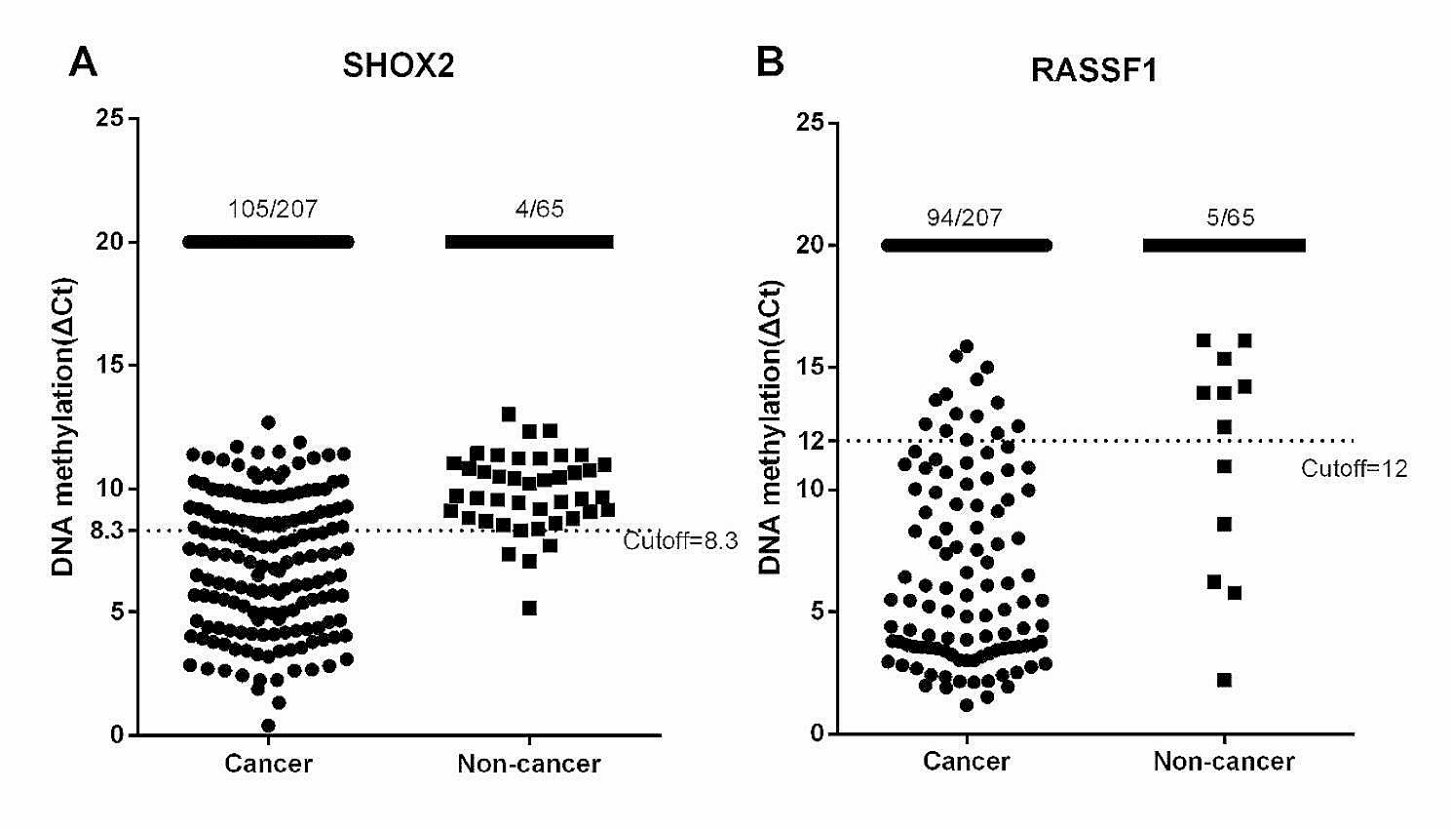
The methylation levels of *SHOX2* and *RASSF1A* in surgical FFPE specimens. A: The methylation levels of *SHOX2* in cancer and non-cancer FFPE specimens. B: The methylation levels of *RASSF1A* in cancer and non-cancer FFPE specimens. Caner group: microinvasive adenocarcinoma (MIA) and invasive adenocarcinoma (IA); Non-cancer group: adenocarcinoma in situ (AIS) and benign lesions

All the specimens were divided into cancer and non-cancer groups. In this case, cancer group contains MIA and IA, while non-cancerous group contains AIS and BC. The ΔCt values of *SHOX2* and *RASSF1A* methylation were utilized to plot scatter plots (Fig. 2). Based on the cutoff values, the positive rate of *SHOX2* and *RASSF1A* methylation were 50.7% (105/207) and 45.4% (94/207) in cancer group. The specificities of *SHOX2* and *RASSF1A* methylation were 93.8% (61/65) and 92.3% (60/65). The sensitivity of LungMe® was 66.2% (137/207), and the specificity was 89.2% (58/65).

**Fig. 3:**
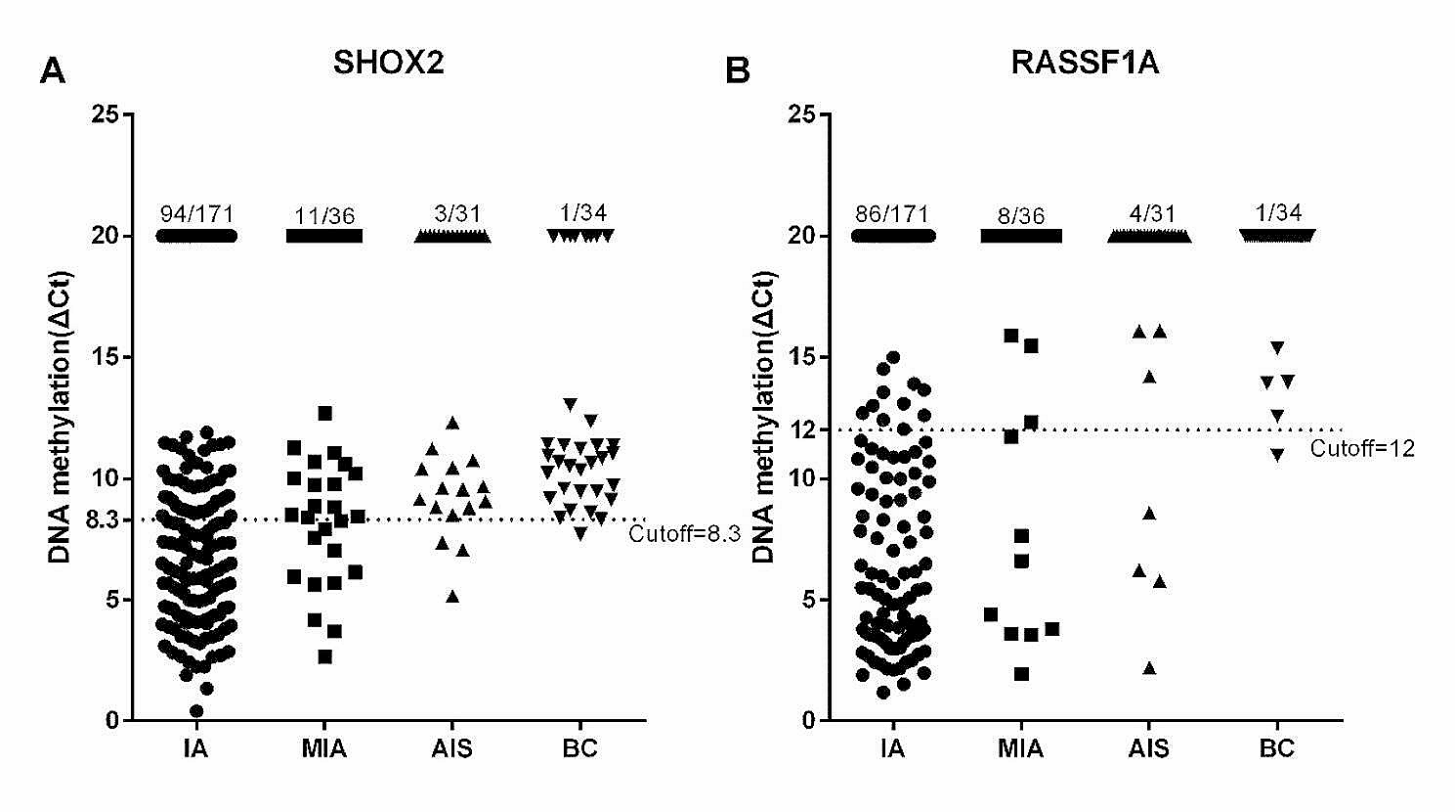
The methylation levels of *SHOX2* and *RASSF1A* in different pathological types of lung adenocarcinoma. A: The methylation levels of *SHOX2* in different pathological types of lung adenocarcinoma. B: The methylation levels of *RASSF1A* in different pathological types of lung adenocarcinoma. AIS: adenocarcinoma in situ; MIA: microinvasive adenocarcinoma; IA: invasive adenocarcinoma; BC: benign control

To further explore the diagnostic value of *SHOX2* and *RASSF1A* methylation in subtypes of lung adenocarcinoma, the methylation levels were calculated in IA, MIA, AIS and BC, respectively. Scatter plots were utilized to represent the ΔCt values of *SHOX2* and *RASSF1A* methylation (Fig. 3). The positive rates of *SHOX2* methylation were 55.0% (94/171), 30.6% (11/36) and 9.7% (3/31) in IA, MIA and AIS patients. Meanwhile, the positive rates of *RASSF1A* methylation were 50.3% (86/171), 22.2% (8/36) and 12.9% (4/31) in IA, MIA and AIS patients. The sensitivities of LungMe® in IA, MIA and AIS patients were 71.3% (122/171), 41.7% (15/36), and 16.1% (5/31). The specificities of *SHOX2*, *RASSF1A* and LungMe® methylation in diagnosing lung adenocarcinoma from benign lesions were 97.1% (33/34), 97.1% (33/34) and 94.1% (32/34) (Table 2).

**Table 2:** The sensitivity and specificity of SHOX2 and RASSF1A methylation in diagnosing lung adenocarcinoma

Methylation indexes	Cutoff	Sensitivity for IA	Sensitivity for MIA	Sensitivity for AIS	Specificity for BC
SHOX2	8.3	55.0% (94/171)	30.6% (11/36)	9.7%(3/31)	97.1% (33/34)
RASSF1A	12	50.3% (86/171)	22.2% (8/36)	12.9%(4/31)	97.1% (33/34)
LungMe®	-	71.3% (122/171)	41.7% (15/36)	16.1%(5/31)	94.1% (32/34)

Note LungMe® is a combination of SHOX2 and RASSF1A methylation

MIA: microinvasive adenocarcinoma; IA: invasive adenocarcinoma

### *Relationship between tumor imaging features and clinicopathological characteristics and methylation in patients with lung adenocarcinoma*

Imaging examinations were utilized to identify various characteristics of tumors, including their size, location, and GGO traits. Tumor traits were divided into pure ground-glass opacity (GGO), mixed ground-glass opacity and solid nodules through imaging examination. The Cavitary-to-Tumor Ratio (CTR) of pure GGO exhibited a value below 0.25, accompanied by a negligible presence of solid components. In contrast, the CTR of mixed GGO fell within the range of 0.25 to 1, whereas solid nodules demonstrated a CTR of 1. Most patients were mixed GGO, accounting for 57.1%, while the other two traits, pure GGO and solid nodules, account for 16.8% and 26.1%, respectively. We found that the tumor traits in patients younger than 65 years have no difference with patients older or equal to 65 years (*p* > 0.05). The number of females was more than that of males, but there was no significant difference (*p* > 0.05). For the tumor size, an overwhelming majority of the tumors were 1–3 cm, accounting for 68.1%. As the size of the tumor increased, there was a gradual progression in the types of nodules observed, transitioning from pure GGO to solid nodules (*p* < 0.001). 78.2% of the patients were classified as stage IA, in which stage the proportion of pure GGO, mixed GGO, and solid nodules were 80.0%, 80.9% and 71.0%. The incidence of solid nodules increased proportionally with the progression of the disease stage (*p* < 0.001). A majority of IA patients, precisely 93.5%, exhibited solid nodules, and only 6.4% of patients with AIS and MIA (*p* < 0.001). According to the World Health Organization’s classification of lung adenocarcinoma, invasive adenocarcinoma can be categorized into three sub-grades. A majority of patients in II grade, accounting for 70.2%. As the stage increased, there was a gradual progression in the types of nodules observed, transitioning from pure GGO to solid nodules. The occurrence of early adenocarcinoma was closely linked to three high-risk factors: lymphnode metastasis, pleural invasion, and airway dissemination. The positive rates of lymph node metastasis, pleural invasion, and airway dissemination are extremely low, whose overall proportions were 3.4%, 11.0% and 7.2%. A significant proportion of patients diagnosed with lymphnode metastasis, pleural invasion and airway dissemination exhibited solid nodules, while being infrequently identified in the pure GGO group (*p* < 0.001). For LungMe® methylation, the positive rates were progressively increased from pure GGO to solid nodules. The positive rate of LungMe® methylation in solid nodule group was 79.0%, which was significantly higher than the rates of the pure GGO and mixed GGO groups, respectively (*p* < 0.05) (Table 3).

**Table 3:** Relationship between tumor imaging features and clinicopathological characteristics in patients with lung adenocarcinoma

Clinicopathological index	*n* = 238	Tumor imaging features [*n* (%)]			*P*
		Pure GGO *n* = 40	Mixed GGO *n* = 136	Solid *n* = 62	
Age (Years)					0.945
<65	116	20(50.0)	65(47.8)	31(50.0)	
≥ 65	122	20(50.0)	71(52.2)	31(50.0)	
Gender					0.138
Male	82	10(25.0)	45(33.1)	27(43.5)	
Female	156	30(75.0)	91(66.9)	35(56.5)	
Tumor size					< 0.001*
≤ 1 cm	59	16(40.0)	36(26.5)	7(11.3)	
>1,≤3 cm	162	24(60.0)	96(70.6)	42(67.7)	
>3 cm	14	0(0.0)	4(2.9)	10(16.1)	
TNM stage					< 0.001*
T0 stage	31	8(20.0)	20(14.7)	3(4.8)	
IA stage	186	32(80.0)	110(80.9)	44(71.0)	
IB stage	11	0(0.0)	5(3.7)	6(9.7)	
II ~ IV stage	10	0(0.0)	1(0.7)	9(3.8)	
Pathological type					< 0.001*
AIS	31	8(20.0)	20(14.7)	3(4.8)	
MIA	36	9(22.5)	26(19.1)	1(1.6)	
IA	171	23(57.5)	90(66 2)	58(93.5)	
IA WHO subtype					< 0.001*
I grade	23	10(43.5)	13(14.4)	1(1.7)	
II grade	120	13(56.5)	74(82.2)	33(56.9)	
III grade	16	0(0.0)	3(3.3)	13(22.4)	
Pleural invasion					0.001*
No	210	37(94.9)	126(93.3)	47(75.8)	
Yes	26	2(5.1)	9(6.7)	15(24.2)	
STAS					< 0.001*
No	218	39(100)	131(97.8)	48(77.4)	
Yes	17	0(0.0)	3(2.2)	14(22.6)	
Lymphnode metastasis					< 0.001*
No	227	39(100)	134(99.3)	54(88.5)	
Yes	8	0(0.0)	1(0.7)	7(11.5)	
LungMe® methylation					0.0011*
Negative	96	21(52.5)	62(45.6)	13(21.0)	
Positive	142	19(47.5)	74(54.4)	49(79.0)	

Note GGO: ground-glass opacity; AIS: Adenocarcinoma in situ; MIA: Microinvasive adenocarcinoma; IA: Invasive adenocarcinoma; IMA: Invasive mucinous adenocarcinoma; STAS: spread through air space. LungMe® is a combination of SHOX2 and RASSF1A methylation

### *Relationship between SHOX2 and RASSF1A methylation and clinicopathological characteristics in patients with lung adenocarcinoma*

The relationship between the *SHOX2* and *RASSF1A* methylation levels and clinicopathological characteristics of all the patients were detected. We found that the positive rate of *SHOX2* methylation in patients younger than 65 years was significantly lower than that in patients older than or equal to 65 years (*p* < 0.05), while there was no significant difference in *RASSF1A* methylation (*p* > 0.05). The positive rate of LungMe® methylation in males was higher than in females (*p* < 0.01). For the tumor imaging features, the positive rates of LungMe® methylation in patients with solid nodules were higher than in the other two groups (*p* < 0.05). The positive rates of *SHOX2*, *RASSF1A* and LungMe® methylation in patients with solid nodules were 62.9%, 58.1% and 79.0%, respectively. The majority of tumor sizes are greater than 1 cm to smaller than 3 cm. The positive rate of LungMe® methylation exhibits an upward trend as tumor size increases (*p* < 0.001). The majority of patients were in IA stage. The methylation positive rates of LungMe® methylation exhibited a gradual increase with the progression of TNM stage, yet experienced a decline in stages II-IV stages, potentially attributable to the error caused by fewer patients (*p* < 0.001). The positive rates of LungMe® methylation exhibit an increase in conjunction with advancements in invasiveness (both *p* < 0.001). For the sub-grades of invasive adenocarcinoma, a majority of patients were in II stage, accounting for 70.2%. As the stage increased, the positive rates of *SHOX2*, *RASSF1A* and LungMe® methylation exhibited an upward trend, with a significant difference observed in LungMe® methylation (*p* < 0.001). The occurrence of early adenocarcinoma is closely linked to three high-risk factors, namely lymphnode metastasis, pleural invasion, and airway dissemination, all of which exhibit relatively low positive rates. Notably, patients with lymphnode metastasis, pleural invasion, and airway dissemination displayed a higher incidence of *SHOX2*, *RASSF1A* and LungMe® methylation compared to those without pleural invasion (*p* < 0.05). *SHOX2*, *RASSF1A* and LungMe® methylation significantly differ among patients with pleural invasion (both *p* < 0.01). *SHOX2* and LungMe® methylation have differences among patients with spread through air space (STAS) (*p* < 0.05). However, no correlation was observed between *SHOX2*, *RASSF1A*, and LungMe® and lymph node metastasis (*p* > 0.05) (Table 4).

**Table 4:** Relationship between SHOX2 and RASSF1A methylation and clinicopathological characteristics in patients with lung adenocarcinoma

Clinicopathological index	*n* = 238	SHOX2 positive [n (%)] *n* = 108	*P*	RASSF1A positive [n (%)] *n* = 98	*P*	LungMe® positive [n (%)] *n* = 142	*P*
Age (Years)			< 0.001*		0.062		0.011*
<65	116	37(31.9)		42(36.2)		60 (51.7)	
≥ 65	122	71(58.2)		56(45.9)		82(67.2)	
Gender			< 0.001*		0.004*		0.001*
Male	82	50(61.0)		44(53.7)		63(76.8)	
Female	156	58(37.2)		54(34.6)		79(50.6)	
Tumor imaging features			0.003*		0.005*		0.001*
Pure GGO	40	13(32.5)		12(30.0)		19(47.5)	
Mixed GGO	136	56(41.2)		50(36.8)		74(54.4)	
Solid	62	39(62.9)		36(58.1)		49(79.0)	
Tumor size			< 0.001*		< 0.001*		< 0.001*
≤ 1 cm	59	13(22.0)		10(16.9)		18(30.5)	
>1,≤3 cm	162	82(50.6)		76(46.9)		109(67.3)	
>3 cm	14	12(85.7)		11(78.6)		13(92.9)	
TNM stage			< 0.001*		< 0.001*		< 0.001*
T0 stage	31	3(9.7)		4(12.9)		5(16.1)	
IA stage	186	90(48.4)		80(43.0)		119(64.0)	
IB stage	11	10(90.9)		10(90.9)		11(100)	
II ~ IV stage	10	5(50.0)		4(40.0)		7(70.0)	
Pathological type			< 0.001*		< 0.001*		< 0.001*
AIS	31	3(9.7)		4(12.9)		5(16.1)	
MIA	36	11(30.6)		8(22.2)		15(41.7)	
IA	171	94(55.0)		86(50.3)		122(71.3)	
IA WHO subtype			0.175		0.251		0.026*
I grade	23	9(39.1)		10(43.5)		12(52.2)	
II grade	120	59(49.2)		65(54.2)		85(70.8)	
III grade	16	11(68.8)		11(68.8)		15(93.8)	
Pleural invasion			0.001*		0.008*		< 0.001*
No	210	88(41.9)		81(38.6)		118(56.2)	
Yes	26	20(76.9)		17(65.4)		24(92.3)	
STAS			0.031*		0.110		0.044*
No	218	96(44.0)		88(40.4)		128(58.7)	
Yes	17	12(70.6)		10(58.8)		14(82.4)	
Lymphnode metastasis			0.276		0.565		0.312
No	227	103(45.4)		94(41.4)		135(59.5)	
Yes	8	5(62.5)		3(37.5)		6(75.0)	

Note LungMe® is a combination of SHOX2 and RASSF1A methylation

GGO: ground-glass opacity; AIS: Adenocarcinoma in situ; MIA: Microinvasive adenocarcinoma; IA: Invasive adenocarcinoma; IMA: Invasive mucinous adenocarcinoma; STAS: spread through air space

### Analysis of high-risk factors for invasiveness of lung adenocarcinoma

All the patients with lung adenocarcinoma and benign lesion were divided into 11 groups through pathological types, pathological subtype, lymph node metastasis, pleural invasion, airway dissemination and TNM stage. Detailedly, the participants were classified into eight grades, namely BN, AIS, MIS, IA1, IA2, IA3, IB, and II-III, according to the TNM staging system. The three grades of IA (IA1, IA2, IA3) were categorized as high or low invasiveness based on the invasiveness of the pathology, resulting in a total of 11 grades. The low invasiveness group were patients with anchorage type or acinar type or papillary type, while the high invasiveness group were patients with micropapillary type or solid type or complex acinar type or pleural invasion or lymph node metastasis or STAS (Table 5).

**Table 5:** The criteria for grouping

Groups	Criteria for grouping	Number	Percentage (%)
1	Benign lesions patients	34	12.8
2	AIS patients in T0 stage	31	11.7
3	MIA patients in IA1 stage	36	13.5
4	IA patients in IA1 stage with low invasiveness	17	6.4
5	IA patients in IA1 stage with high invasiveness	0	0.0
6	IA patients in IA2 stage with low invasiveness	85	32.0
7	IA patients in IA2 stage with high invasiveness	7	2.6
8	IA patients in IA3 stage with low invasiveness	19	7.1
9	IA patients in IA3 stage with high invasiveness	18	6.8
10	IA patients in IB stage	10	3.8
11	Patients in II + III stages	10	3.8

Note The low invasiveness groups: anchorage type or acinar type or papillary type; the high invasiveness groups: micropapillary type or solid type or complex acinar type or pleural invasion or lymph node metastasis or spread through air space

Multivariate linear regression was performed to analyze the hazardous factors affecting the invasiveness of lung adenocarcinoma. Prior to the surgical procedure, pertinent information including age, gender, methylation level, tumor size, and imaging features was available and subsequently utilized for conducting univariate analysis. Univariate analysis results indicated that when increased by one year, the grade increased by 0.061 (*p* < 0.05). For gender, the grades of males were lower by 0.142 than females, but the difference was not statistically significant (*p* > 0.05). Tumor size increased by one year, and the grade increased by 0.987 (*p* < 0.05). LungMe® methylation positively increased by 2.857 on grade compared to negative methylation (*p* < 0.05). The grade of patients with mixed GGO was lower by 0.454 than pure GGO, but the difference was not statistically significant (*p* > 0.05). The grade of patients with solid nodules was higher by 1.221 than pure GGO (*p* < 0.05). Age, tumor size and solid nodules, which were statistically significant in univariate analysis, were included in the multivariate regression analysis. Through multivariate analysis, the age, methylation level, and tumor size were identified as independent hazardous factors influencing the invasiveness of lung adenocarcinoma (*p* < 0.05) (Table 6).

**Table 6:** The hazardous factors influencing the invasiveness of lung adenocarcinoma

Variables	Univariate analysis		Multivariate analysis	
	HR (95% CI)	*P*	HR (95% CI)	*P*
Age (Years)	0.061(0.032,0.090)	<0.001	0.031(0.006,0.056)	0.015
Gender(Male/Female)	-0.142(-0.851,0.567)	0.693	-	-
Tumor size	0.987(0.694,1.280)	<0.001	0.549(0.270,0.827)	<0.001
LungMe methylation	2.857 (2.245,3.470)	<0.001	2.398 (1.806,2.990)	<0.001
Tumor imaging features				
Mixed GGO	-0.454(-1.146,0.238)	0.198	-	-
Solid nodules	1.221(0.474,1.968)	0.001	0.887(0.234,1.540)	0.008

Note HR, hazard ratio; 95% CI: 95% Confidence Interval. GGO: ground-glass opacity, Mixed GGO: 0.25 < CTR < 1; Solid nodules: CTR = 1. *P < 0.005

**Table 7:** Relationship between surgical procedure and risk factors associated with invasiveness of lung adenocarcinoma

Index	*N* = 235	Wedge resection(35)	Segmentectomy(77)	Lobectomy(123)	*P*
Tumor size					< 0.0001*
≤ 2 cm	178	28(15.7)	70(39.3)	80(44.9)	
> 2 cm	57	7(12.3)	7(12.3)	43(75.4)	
Tumor imaging features					0.011*
CTR ≤ 0.5	173	25(14.5)	66(38.2)	82(47.4)	
CTR > 0.5	62	10(16.1)	11(17.7)	41(66.1)	
LungMe® methylation					< 0.0001*
Positive	141	20(14.2)	33(23.4)	88(62.4)	
Negative	94	15(16.0)	44(46.8)	35(37.2)	

Note LungMe® is a combination of SHOX2 and RASSF1A methylation. CTR: Cavitary-to-tumor ratio

**Fig. 4:**
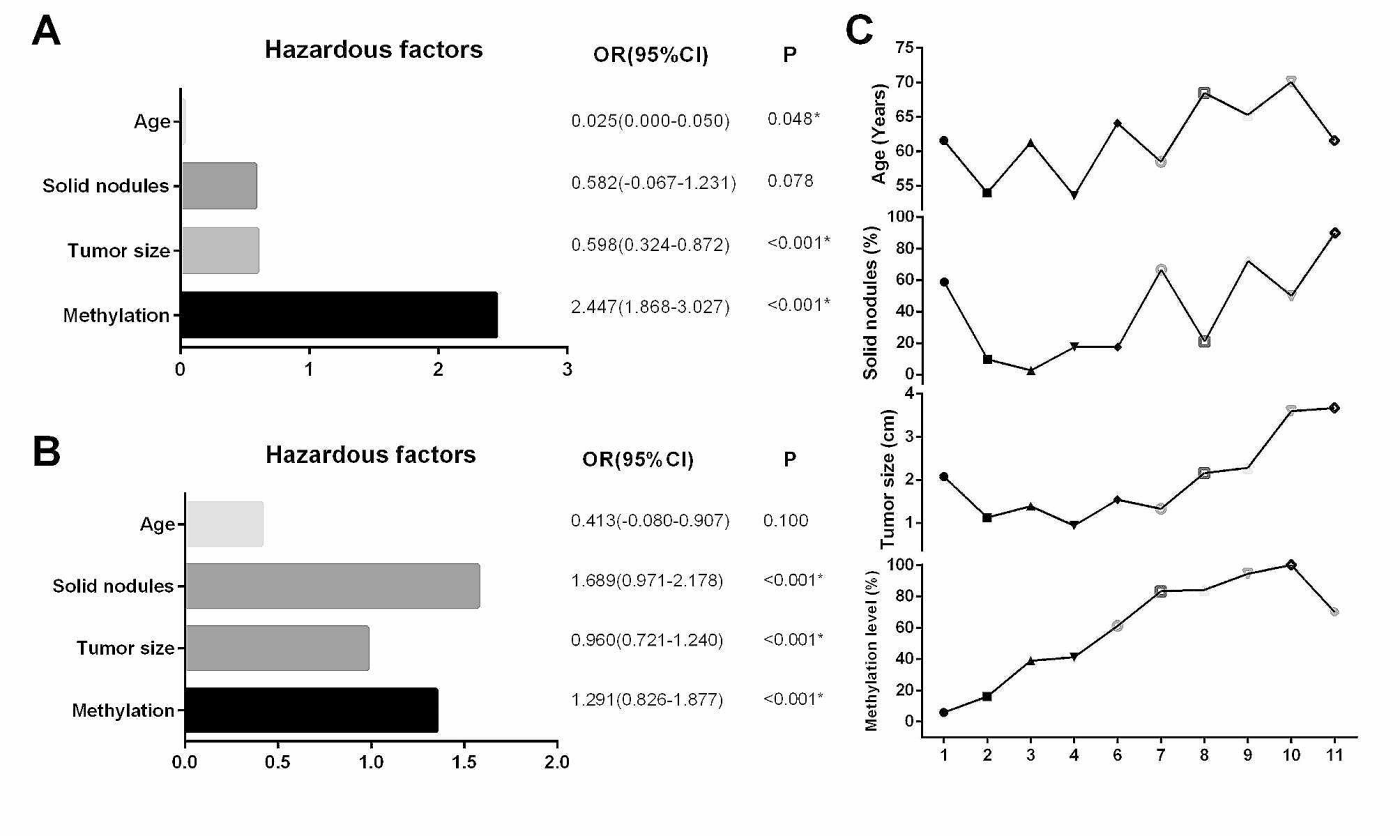
Analysis of high-risk factors for invasiveness of lung adenocarcinoma. A: The hazardous factors of benign and malignant lung adenocarcinoma. B: The hazardous factors influencing the invasiveness of lung adenocarcinoma. C: The average levels of age and tumor size, the proportions of methylation level and solid nodule in lung adenocarcinoma. HR, hazard ratio; 95% CI: 95% Confidence Interval. *P < 0.005

The inclusion of patients with benign lesions revealed that LungMe® methylation positive exerted a significant influence on the invasiveness of lung adenocarcinoma, as indicated by a hazard ratio (HR) value of 2.447 (Fig. 4A). Including patients without benign lesions revealed that solid nodules had the greatest impact on the invasiveness of lung adenocarcinoma, with an HR value of 1.689, followed by a positive LungMe® methylation of 1.291. However, age does not affect the grade when the patient has already identified the tumor (Fig. 4B). In addition, the average levels of age and tumor size, the positive rate of methylation level and proportions of solid nodules in lung adenocarcinoma were shown in Fig. 4C. It can be found that as the lung adenocarcinoma invasiveness increases, there is an upward trend in both the four indexes, especially methylation level.

### Relationship between surgical procedure and risk factors associated with invasiveness of lung adenocarcinoma

Three surgical methods were wedge resection, segmentectomy and lobectomy, with lobectomy representing over 50% of the cases. A total of 178 patients were diagnosed with tumors measuring less than 2 cm, whereas 57 patients presented tumors exceeding 2 cm in size. The treatment approach for patients with tumors smaller than 2 cm primarily involved segmentectomy and lobectomy, whereas tumors larger than 2 cm were predominantly subjected to lobectomy (*p* < 0.05). Similar to the situation of tumor size, patients presenting a CTR below 0.5 predominantly underwent segmentectomy and lobectomy, while the majority of patients with a CTR exceeding 0.5 underwent lobectomy (*p* < 0.05). A total of 62.4% of patients exhibiting positive methylation underwent lobectomy, and patients with lobectomy were more than segmentectomy. In contrast, among patients with negative methylation, the majority of patients underwent segmentectomy more than lobectomy (*p* < 0.05)（(Table 7).

## Discussion

The widespread adoption of low-dose computed tomography (LDCT) has facilitated the early detection of lung adenocarcinoma. Nevertheless, LDCT’s high sensitivity may lead to the identification of numerous, smaller early lesions, necessitating the assessment of benign and malignant nodules. Increasing evidence indicated that DNA methylation alteration is regarded as an early prognosticator of cancer and can be identified during the initial phases of tumorigenesis [15–18]. The combination detection of *SHOX2* and *RASSF1A* methylation has been utilized in diagnosing lung cancer, with a sensitivity of 71.5–96.0%, and a specificity of 82.3–100% [13, 19].

In this study, *SHOX2* and *RASSF1A* gene methylation were utilized to diagnose early lung adenocarcinoma, while surgical tissue samples were employed to mitigate the potential impact of sampling variability. The majority of prior investigations were conducted using bronchoalveolar lavage fluid, thus necessitating the establishment of novel threshold values in tissue specimens. To ascertain the threshold values for *SHOX2* and *RASSF1A*, two distinct grouping methodologies were employed to draw ROC curves, resulting in similar cutoff values of 8.3 and 12.0. Considering prior research, the cutoff value of *SHOX2* was 7.5 in surgical tissues, which was influenced by the inclusion of a substantial number of granulomas [14]. However, in the current study, surgical samples of tuberculosis were deliberately excluded from the initial stage of the experimental data, resulting in improved specificity and a revised cutoff value of 8.3. The combined AUCs of *SHOX2* and *RASSF1A* were 0.814 and 0.770, respectively, exceeding the threshold of 0.75, indicating that the two methods all have diagnostic values. In this study, the sensitivity and specificity of LungMe® methylation in diagnosing lung adenocarcinoma were 66.2% and 94.1%. Our results were consistent with the previous research that the sensitivity and specificity of LungMe® methylation were 69.6% and 97.4 [13]. A prior investigation demonstrated that the diagnostic sensitivity of *SHOX2* and *RASSF1A* combined methylation in lung adenocarcinoma was 52.5%, whereas cytology exhibited a sensitivity of 13.3%. Furthermore, the combined utilization of methylation and cytology yielded a sensitivity of 55.8% [20]. The sensitivity of DNA methylation detection in diagnosing lung cancer, particularly LUAD, was found to be higher compared to cytology detection, while also compensating for the limitations of cytology [21]. Methylation detection is a molecular method that utilizes PCR to greatly enhance the methylation signal by a factor of 1 million, exhibiting remarkable sensitivity and amplification specificity. In comparison to cytology, methylation demonstrates significantly higher sensitivity while maintaining equivalent specificity. Nevertheless, this study did not undertake a comparative analysis between cytology and methylation, which will be addressed in the subsequent article.

The classification of early adenocarcinoma was based on the extent of invasion, resulting in the categorization of IA, MIA, and AIS. The reclassification of AIS prompts an inquiry into the necessity of surgical intervention, specifically determining when surgery is warranted and when surgery is unnecessary for AIS. From a pathological diagnostic standpoint, AIS and MIA exhibit overlapping characteristics. AIS can be likened to a dormant intruder within the body, and it becomes imperative to ascertain the moment when this intruder may become active. Our contemplation revolves around the potential utility of methylation as a reliable triage indicator, and whether positive methylation can serve as a determinant for the impending activation of the intruder. The sensitivities of LungMe® in IA, MIA and AIS patients were 71.3% (122/171), 41.7% (15/36), and 16.1% (5/31), which was very consistent with our expectation. The specificities of LungMe® methylation in diagnosing lung adenocarcinoma from benign lesions was 94.1% (32/34). The methylation of LungMe® demonstrates significant diagnostic efficacy in the identification of lung adenocarcinoma, while also exhibiting varying discriminatory potential across distinct pathological stages of early lung adenocarcinoma. Research has demonstrated a positive association between the combined methylation levels of *SHOX2* and *RASSF1A* and the expression of Ki-67 in early-stage lung adenocarcinoma [22]. Ki-67 is known to be involved in the proliferation of cancer cells, with higher values indicating accelerated tumor growth and development, ultimately leading to a poorer prognosis for patients [23–25]. It can be inferred that individuals who test positive for *SHOX2* and *RASSF1A* methylation may experience more rapid tumor progression. Our results indicated that patients with IA had the highest sensitivity compared to MIA and AIS, which was consistent with the previous findings. Also, the association between *RASSF1A* and the heightened invasiveness of lung cancer has been reported. Suppression of the *RASSF1A* gene facilitated the invasion and migration of lung cancer cells [26]. Thus, there is a positive correlation between the invasiveness of the tumor and the rate of LungMe® methylation, with higher invasiveness resulting in a higher positive rate. The presence of positive LungMe® methylation in patients with AIS suggests a potential for invasion, which could progress to MIA, thereby recommending surgical intervention. This is the first time to propose that positive LungMe® methylation in patients with AIS may illustrate a risk for invasion.

Furthermore, the pathological characteristics associated with high invasiveness include tumor size, TNM stage, pathological type, lymph node metastasis, pleural invasion and STAS. Imaging serves as the primary clinical examination for discerning between benign and malignant conditions prior to surgical intervention, and its significance as a preoperative indicator is of utmost importance. Prior to assessing methylation, we initially explored the relationship between imaging features and highly aggressive attributes. A Previous study found that solid components are a negative prognostic factor in lung adenocarcinoma [27]. Especially, we discovered that a LungMe® methylation positive status was observed in 79% of solid nodules patients, which is consistent with prior research. The methylation of LungMe® exhibited correlations with lung adenocarcinoma high-risk factors, pleural invasion and STAS, except for lymph node metastasis. Despite a lack of statistical significance, patients with positive lymph node metastasis exhibited a higher degree of LungMe® methylation. The methylation level of *SHOX2* was found to be significantly higher in patients with positive STAS, whereas no significant association was observed between the methylation level of *RASSF1A* and STAS. Considering the different pathological subtypes of adenocarcinoma, invasive adenocarcinoma can be categorized into three sub-grades according to the World Health Organization’s classification. The anchorage type was designated in I grade, while the acinar and papillary types were classified in II grade. Additionally, the solid, micropapillary, and complex acinar types were categorized in III grade. Previous research illuminated that lung adenocarcinoma at TNM I stage with minimal solid or micropapillary have higher invasiveness, thus predicting poor prognosis [28]. Consistent with the findings, we found that LungMe® methylation positive rates exhibited a gradual increase with the progression of IA subtype. The methylation rate in III grade, specifically in the subtype characterized by solid, micropapillary, and complex acinar features, was determined to be 93.8%. LungMe® methylation exhibited correlations with all pathological characteristics associated with high invasiveness, with the exception of lymph node metastasis, indicating a close association between methylation and the attributes of heightened invasiveness.

The controversy surrounding preoperative lobectomy or segmentectomy persists within academic discourse. Segmentectomy is a comparatively smaller surgical procedure than lobectomy for treating small NSCLC lesions. However, due to the presence of pathologically aggressive tumor features, there is a potential risk of positive surgical margins for cancer and subsequent recurrence. The research of JCOG0802/WJOG4607L also identified a comparatively elevated incidence of local recurrence associated with segmentectomy in contrast to lobectomy [29]. Consequently, the careful selection of appropriate surgical methods holds significant potential in enhancing the recurrence outcomes for early lung adenocarcinoma [30].

We want to make an initial assessment of the invasiveness of nodules based on the available information prior to surgery, in order to determine the most suitable surgical procedure and treatment approach. All the lung adenocarcinoma and benign lesions patients were categorized into 11 cohorts based on the invasiveness, and an analysis was conducted of the risk factors influencing the invasiveness of lung adenocarcinoma. Incorporating patients with benign lesions, the positive rate of LungMe® methylation was increasing, which serves as compelling evidence for assessing the level of invasiveness. However, the observed decline in group 11 may be attributed to the limited patient sample size. We also found that as the lung adenocarcinoma invasiveness increases, there is an upward trend in all the indexes, especially LungMe® methylation level. Consequently, wedge resection emerges as a viable treatment option for AIS and MIA. The research of JCOG0802/WJOG4607L first proposed that segmentectomy exhibits greater efficacy in terms of overall survival for early-stage lung cancer. They ultimately found that segmentectomy ought to be considered as the prevailing surgical approach for patients diagnosed with peripheral non-small cell lung cancer, characterized by a diameter of ≤ 2 cm and a CTR exceeding 0.5 [29]. When a patient is diagnosed with a tumor, early diagnostic indicators such as size, solid nodule (CTR = 1) and LungMe® methylation, exhibit a strong correlation with the invasiveness of the tumor, thereby enabling the establishment of a predictive model. Specifically, we incorporated a new highly sensitive indicator, LungMe® methylation, which was novel to this study. The calculated score not only assesses the invasiveness of the tumor but also offers supplementary information for determining the appropriate surgical approach based on tumor size.

## Conclusion

LungMe® methylation can be used as an early detection indicator of lung adenocarcinoma, as it is associated with highly aggressive subtypes. The potential risk factors for the invasiveness of lung adenocarcinoma include age, methylation level, and tumor size. The *SHOX2* and *RASSF1A* methylation levels, tumor size and CTR values could predict the invasiveness of the tumor prior to surgery, thereby providing guidance for the surgical procedure.

## Data Availability

All data can be obtained from the corresponding author.

## Abbreviations

NSCLC: Non-small cell lung cancer
SHOX2: Short stature homeobox 2
RASSF1A: Ras-association domain family member 1 A
MS-PCR: Methylation-specific PCR
FFPE: Formalin fixed paraffin-embedded
AIS: Adenocarcinoma in situ
MIA: Microinvasive adenocarcinoma
IA: Invasive adenocarcinoma
BC: Benign lesions control
NMPA: National Medical Products Administration
IVD: In vitro diagnostic
ROC: Receiver operator characteristic
AUC: Areas under the curve
GGO: Ground-glass opacity
CTR: Cavitary-to-tumor ratio
STAS: Spread through air space
HR: Hazard ratio
LDCT: Low-dose computed tomography
LUAD: Lung adenocarcinoma
